# Viscoelastic-Assisted Rescue of Unintended Free Internal Limiting Membrane Flaps During Macular Hole Surgery: A Two-Case Report

**DOI:** 10.3390/jcm15197392

**Published:** 2026-09-23

**Authors:** Keisuke Minami, Ryoji Yanai, Yoshinori Mitamura

**Affiliations:** Department of Ophthalmology, Institute of Biomedical Sciences, Tokushima University Graduate School, 3-18-15 Kuramoto, Tokushima 770-8503, Japan; minami.keisuke2@tokushima-u.ac.jp (K.M.); ymita@tokushima-u.ac.jp (Y.M.)

**Keywords:** macular hole, internal limiting membrane, free flap transplantation, ophthalmic viscosurgical device, vitrectomy

## Abstract

**Background/Objectives:** Manipulation of an internal limiting membrane (ILM) flap can be technically challenging during macular hole (MH) surgery, particularly in highly myopic eyes with fragile ILM. Although ophthalmic viscosurgical devices (OVDs) have been used to stabilize inverted ILM flaps, their use for the rescue of unintended free-floating ILM flaps has been less well described. This study aimed to describe a perfluorocarbon liquid (PFCL)-free, viscoelastic-assisted rescue technique for the capture, transfer, and stabilization of unintended free ILM flaps during MH surgery. **Methods:** Two highly myopic eyes with MH underwent 27-gauge pars plana vitrectomy with an ILM flap technique. When the ILM became unintentionally detached and formed a free-floating flap, a dispersive OVD was delivered through a blunt-tip cannula to capture the flap. The OVD and attached flap were manipulated as a single unit and repositioned over the MH without direct forceps grasping. An additional OVD was applied over the flap for stabilization before fluid–air exchange and 20% sulfur hexafluoride gas tamponade. **Results:** MH closure was confirmed via optical coherence tomography on the first postoperative day in both eyes and was maintained through the final follow-up at 5 months in Case 1 and at 1 month in Case 2. Best-corrected visual acuity was 20/33 (0.22 logMAR) at final follow-up in both eyes, compared with 20/40 (0.30 logMAR) preoperatively. No procedure-related intraoperative or postoperative complications were observed during the available follow-up periods. **Conclusions:** This viscoelastic-assisted technique may provide a practical rescue approach for unintended free-floating ILM flaps during MH surgery without the need for direct forceps grasping or PFCL. Larger comparative studies with longer follow-up are needed to evaluate its reproducibility, safety, and clinical utility.

## Simple Summary

A viscoelastic-assisted technique allowed manipulation and placement of unintended free ILM flaps without the need for direct forceps grasping or perfluorocarbon liquid during macular hole surgery.

## 1. Introduction

As it enhances anatomical closure rates and reduces the risk of reopening, internal limiting membrane (ILM) peeling is widely established as a standard technique in macular hole (MH) surgery [1]. However, successful closure can still be difficult to achieve in challenging cases, such as highly myopic eyes [2], chronic MHs [3], and large MHs [4]. In such cases, various ILM flap techniques have been developed to improve anatomical outcomes.

The inverted ILM flap technique, first described by Michalewska et al. [5], has become an important surgical option for difficult MHs. Recent evidence has further supported the role of ILM flap techniques in MH surgery; for example, a 2025 individual participant data meta-analysis of 13 trials involving 792 eyes demonstrated that ILM flap techniques were associated with higher primary closure rates and better postoperative visual acuity than conventional ILM peeling, with greater benefits observed in larger MHs. [6] In highly myopic eyes, a recent case–control study also reported a lower rate of non-closure with ILM flap techniques than with ILM peeling [7]. These findings are particularly relevant because high myopia poses additional surgical challenges related to axial elongation, posterior staphyloma, and altered posterior pole anatomy.

Despite these advances, maintaining the ILM flap in the intended position remains technically challenging. Several adjunctive techniques have therefore been developed to stabilize the flap during surgery. Recent studies have described the use of ophthalmic viscosurgical devices (OVDs) to stabilize inverted ILM flaps; for example, Fan et al. reported a “viscoelastic agent pool” technique for stabilizing inverted ILM flaps in eyes with MH-associated retinal detachment [8], while Li et al. recently described OVD-assisted press injection for the stabilization of a sectorial inverted ILM flap [9].

However, these techniques primarily focus on the stabilization of an inverted ILM flap that remains available for placement over the MH. Intraoperatively, particularly in highly myopic eyes with fragile ILMs, the flap may become completely detached during manipulation and form a free-floating ILM flap. Free ILM flap capture, transfer, and placement are technically challenging, and direct manipulation with vitreoretinal forceps may carry a risk of inadvertent retinal contact and injury. Free ILM transplantation has also been described as a treatment option for persistent or recurrent MHs after previous ILM removal [10,11,12].

In this report, we describe an OVD-assisted technique as a rescue maneuver for unintended free-floating ILM flaps during MH surgery in two highly myopic eyes. Unlike previously described OVD-assisted techniques for stabilizing inverted ILM flaps, the present technique uses a dispersive OVD to capture, transfer, and stabilize an already detached free ILM flap without the need for direct forceps grasping or perfluorocarbon liquid (PFCL).

## 2. Methods

Two patients underwent three-port 27-gauge pars plana vitrectomy using the CONSTELLATION^®^ Vision System (Alcon Laboratories, Inc., Fort Worth, TX, USA). The residual vitreous cortex was visualized using triamcinolone acetonide and subsequently removed. The ILM was stained with Brilliant Blue G (ILM Blue^®^; D.O.R.C., Zuidland, The Netherlands) and peeled from the macular region. No myopic foveoschisis, macular detachment, or other relevant myopic macular abnormalities were identified in either study eye. Both eyes had posterior staphyloma. Given the high axial myopia and posterior staphyloma in both eyes, we selected an ILM flap technique because these anatomical features can make MH closure more challenging. During the period in which these cases were encountered, unintended free ILM flap formation occurred in two MH surgeries. The OVD-assisted rescue technique was applied in both cases, and both are included in this report.

When the ILM became detached, thus forming a free flap, a dispersive ophthalmic viscosurgical device (OVD) (Viscoat^®^; Alcon Laboratories, Inc., Fort Worth, TX, USA) was used to capture and reposition the flap. The infusion cannula was positioned such that the infusion flow was directed away from the macular region, and the intraocular pressure was maintained at 15 mmHg during flap manipulation. The OVD was extruded approximately two optic disc diameters beyond the tip of a 27-gauge blunt-tip cannula and directed toward the free-floating ILM flap to capture it. The OVD and attached ILM flap were then manipulated as a single unit and positioned over the MH, taking care not to introduce the OVD into the MH or between the flap and the retinal surface. Excessive extrusion of the OVD was avoided to facilitate manipulation of the OVD and flap as a single unit. Before fluid–air exchange, an additional OVD was gently applied over the flap, covering an area approximately three times the diameter of the MH, in order to stabilize the flap (Figure 1 and Figure 2). The OVD overlying the ILM flap was intentionally retained during fluid–air exchange; no direct attempt was made to aspirate the OVD from the macular area. Fluid–air exchange was subsequently performed using a backflush needle positioned over the optic disc to minimize the risk of aspirating the OVD or ILM flap. The vitreous cavity was then filled with 20% sulfur hexafluoride (SF6) gas. The surgical procedure is demonstrated in Supplemental Digital Content S1. Patients were instructed to maintain a prone position until MH closure was confirmed via OCT on the first postoperative day. After confirmation of MH closure, patients were allowed to assume any position except the supine position until the intraocular gas had completely resolved. The exact OVD volume in milliliters and the dimensions of the unintended free ILM flaps were not quantitatively recorded intraoperatively; therefore, no retrospective numerical estimates were introduced.

As transverse optical coherence tomography (OCT) measurements may underestimate the MH size in highly myopic eyes due to ocular magnification from axial elongation, corrected MH diameters were additionally calculated using linear scaling based on axial length, as follows: corrected MH diameter = measured MH diameter × axial length/24.0 mm [13].

## 3. Case Reports

### 3.1. Case 1

A 78-year-old man presented with a full-thickness MH in his right eye (Figure 3A). He had undergone phacoemulsification and intraocular lens (IOL) implantation in the right eye 10 years previously. Four months before the onset of the MH, he underwent pars plana vitrectomy and intrascleral IOL fixation for IOL dislocation. His ocular history was also significant for bilateral glaucoma, and his systemic medical history included angina pectoris and benign prostatic hyperplasia. The right eye was pseudophakic, with a spherical equivalent of −7.5 D and an axial length of 28.92 mm. The duration from symptom onset to surgery was 8 weeks. The preoperative best-corrected visual acuity (BCVA) was 20/40 (0.30 logMAR), and the intraocular pressure (IOP) was 7.0 mmHg. The minimum hole diameter measured 354 μm on OCT and 427 μm after correction for axial length. During ILM peeling, the ILM flap became detached from the forceps and formed a free-floating flap. The flap was captured and repositioned over the MH using the OVD-assisted technique described above. On the first postoperative day, the IOP was 7.3 mmHg, and MH closure was confirmed via OCT (Figure 3B). At 2 weeks postoperatively, it was confirmed via OCT that the MH remained closed (Figure 3C). At the final follow-up (5 months after surgery), the MH remained closed, BCVA was 20/33 (0.22 logMAR), and IOP was 6.3 mmHg. The OCT results indicated persistent disruption of the external limiting membrane (ELM) and ellipsoid zone (EZ) beneath the fovea (Figure 3D).

### 3.2. Case 2

A 62-year-old woman presented with a full-thickness MH in her right eye (Figure 3E). The right eye was phakic with cataract, with a spherical equivalent of −13.5 D and an axial length of 29.31 mm. Posterior staphyloma was present. She had no history of previous ocular surgery or other ocular diseases. Her systemic medical history included breast cancer, Graves’ disease, and asthma. The duration from symptom onset to surgery was 3 weeks. The preoperative best-corrected visual acuity (BCVA) was 20/40 (0.30 logMAR), and the intraocular pressure (IOP) was 16.6 mmHg. The minimum hole diameter measured 229 μm on OCT and 280 μm after correction for axial length. During ILM peeling, the fragile ILM tore and became a free-floating flap. The flap was captured and repositioned over the MH using the OVD-assisted technique described above. On the first postoperative day, the IOP was 8.0 mmHg, and MH closure was confirmed via OCT (Figure 3F). At 2 weeks postoperatively, the OCT results confirmed that the MH remained closed (Figure 3G). At the final follow-up (1 month after surgery), the MH remained closed, BCVA was 20/33 (0.22 logMAR), and IOP was 16.0 mmHg. The OCT results demonstrated preservation of the ELM and EZ beneath the fovea (Figure 3H).

## 4. Discussion

In this report, we achieved successful MH closure in two highly myopic eyes using the described viscoelastic-assisted technique, which was maintained through the available follow-up. The final BCVA was 20/33 (0.22 logMAR) in both eyes, compared with 20/40 (0.30 logMAR) preoperatively. No procedure-related complications, including retinal injury, were noted.

Free ILM flaps may arise inadvertently during ILM peeling, or may be intentionally used in patients with recurrent MH following prior ILM removal. Moreover, eyes with high myopia pose particular surgical challenges due to axial elongation, posterior staphyloma, and altered contour of the posterior pole, which can make ILM manipulation technically demanding. In such eyes, the ILM is often fragile and may fragment during peeling, leading to an unintended free ILM flap. The described viscoelastic-assisted technique may therefore be particularly useful in situations requiring the controlled capture and stabilization of a free ILM flap. Importantly, the present technique differs from previously reported OVD-assisted techniques for inverted ILM flaps, in that the OVD is used as a rescue maneuver to capture, transfer, and stabilize an already detached, free-floating ILM flap.

Previously described techniques for managing free ILM flaps include direct manipulation with vitreoretinal forceps, ILM translocation, and stabilization using PFCL [10,11,12]. Direct forceps manipulation requires careful handling to minimize unintended contact with the retinal surface. PFCL may facilitate the stabilization of a free ILM flap; however, retained subfoveal PFCL has been reported as a potential complication of vitreoretinal surgery and has been associated with secondary macular hole formation [14,15]. These considerations provided the rationale for exploring an alternative approach using an OVD in the present cases.

The presented viscoelastic-assisted technique enables the capture and transfer of a floating ILM flap without the need for direct grasping with vitreoretinal forceps. In the present two cases, the free ILM flap was successfully repositioned using a commercially available OVD without direct forceps grasping or PFCL. However, given the limited number of cases, the short follow-up in Case 2, and the absence of a comparative control group, these observations do not establish greater safety, reduced retinal trauma, or superiority over conventional forceps- or PFCL-assisted techniques.

Viscoat^®^ was selected for its dispersive and viscous properties, which may facilitate adherence to and controlled movement of a free-floating ILM flap, thereby allowing the flap to be manipulated without direct grasping. A crucial technical aspect of this procedure is the use of a blunt-tip cannula, which enables controlled delivery of the OVD into the vitreous cavity. Excessive intraocular fluid currents should be avoided, because irrigation flow may readily displace the floating flap. Furthermore, residual OVD may theoretically interfere with close apposition between the ILM flap and the retinal surface. Therefore, careful control of the amount and direction of OVD injection is important, and the potential effects of residual OVD warrant further investigation.

## 5. Conclusions

The viscoelastic-assisted technique described in this report may provide a practical rescue approach for the capture, transfer, and stabilization of an unintended free-floating ILM flap during MH surgery. The use of a dispersive OVD allowed the detached ILM flap to be repositioned over the MH without the need for direct forceps grasping or PFCL in the two cases described here. However, the small number of cases, the limited follow-up duration (particularly in Case 2), and the absence of a comparative control group preclude conclusions regarding the safety or superiority of this technique over other methods. Further studies involving larger cohorts, longer follow-up, and comparison with conventional techniques are warranted to evaluate its reproducibility, safety, and clinical utility.

## Figures and Tables

**Figure 1:**
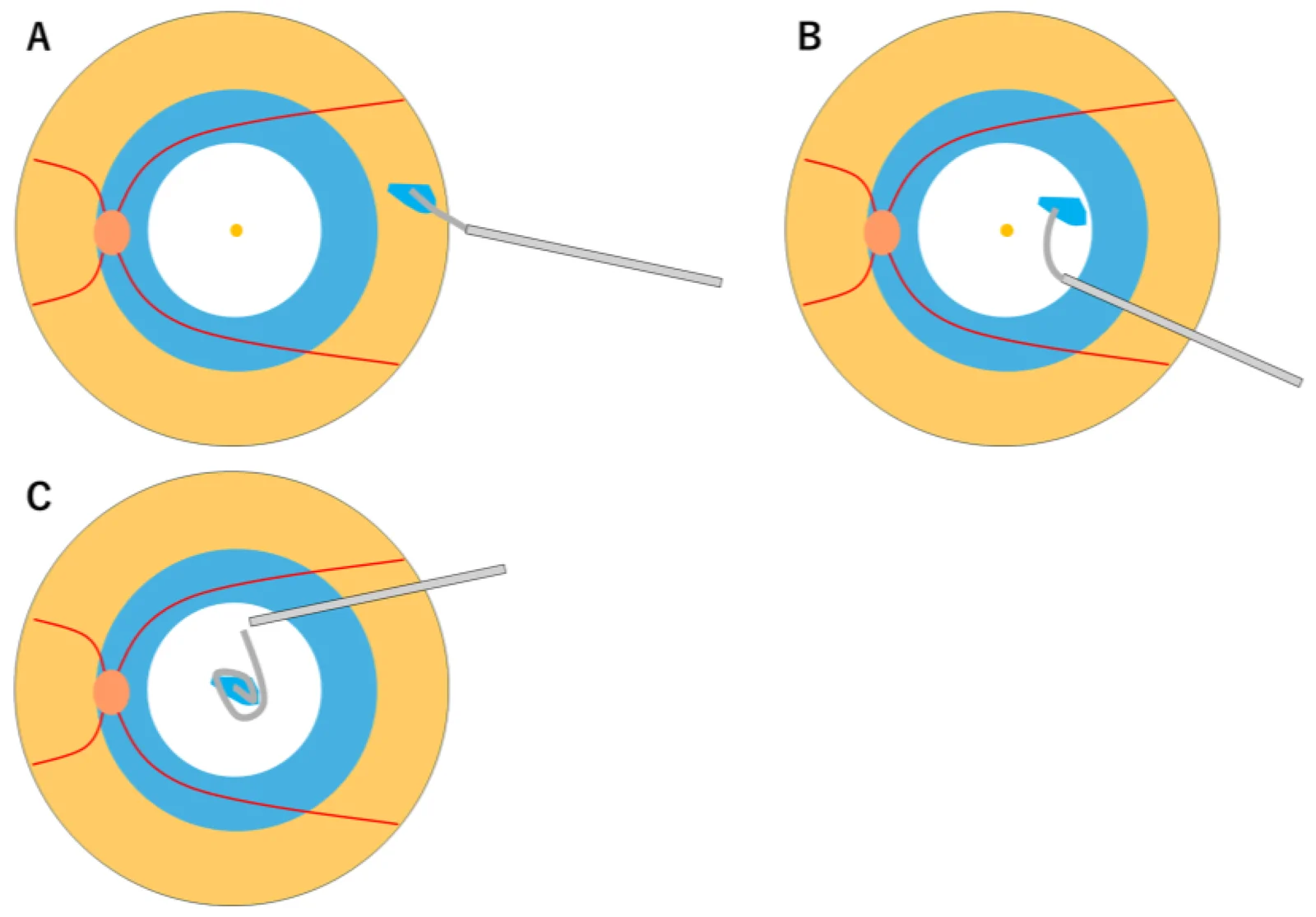
Schematic representation of the surgical procedure. (**A**) After staining the macular area with Brilliant Blue G (BBG), the internal limiting membrane (ILM) surrounding the macular hole was peeled. Viscoat was delivered toward the free ILM flap using a 27-gauge blunt-tip cannula. (**B**) The free ILM flap was guided toward the macular hole while adhering to the viscoelastic material. (**C**) Additional Viscoat was injected to stabilize and secure the free ILM flap over the macular hole.

**Figure 2:**
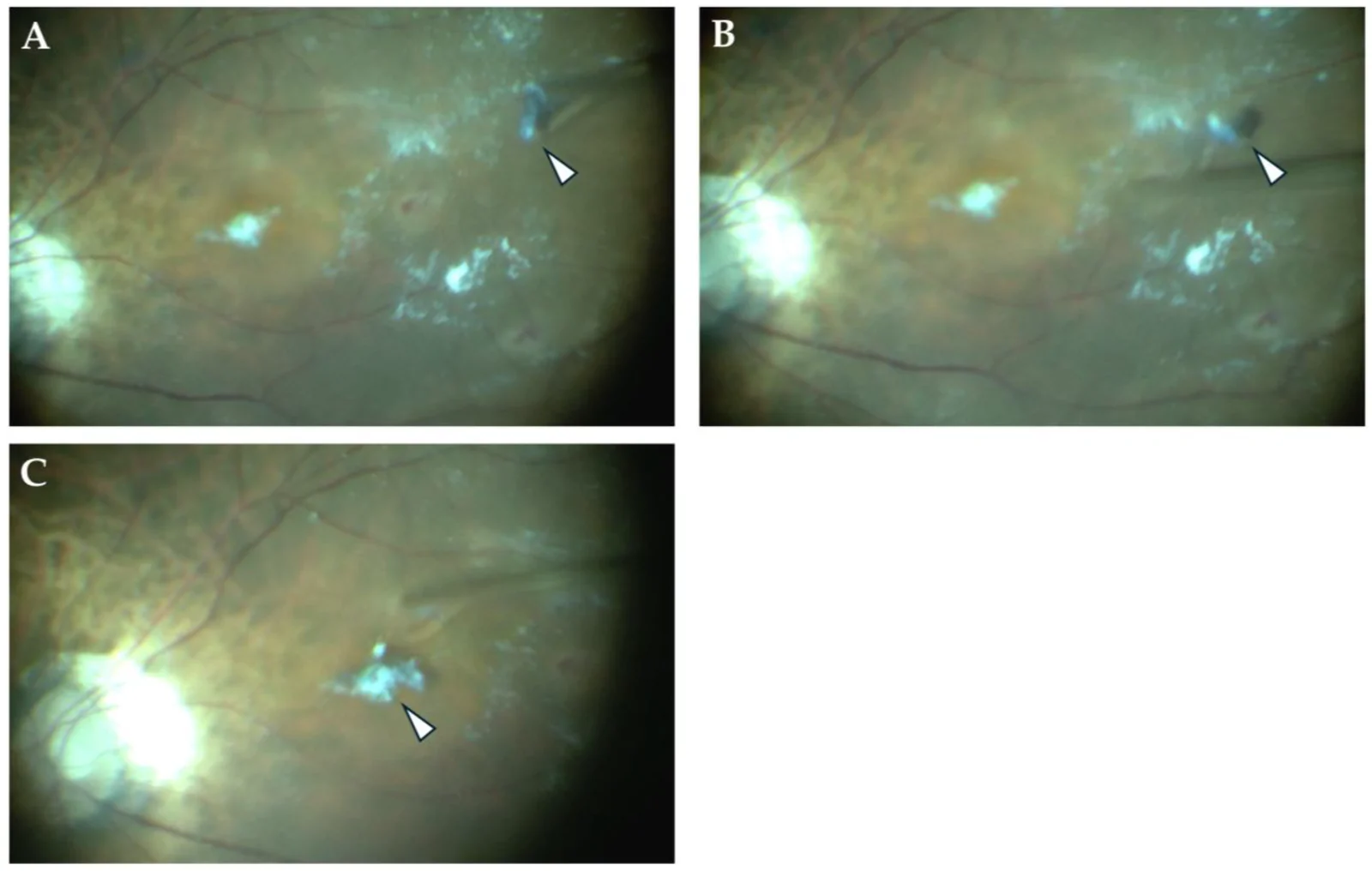
Intraoperative fundus photographs illustrating the viscoelastic-assisted free ILM flap technique. (**A**) Viscoat injected through a 27-gauge blunt-tip cannula adheres to the free ILM flap (arrowhead). (**B**) The free ILM flap (arrowhead) is advanced toward the macular hole while adhering to the viscoelastic material. (**C**) The free ILM flap (arrowhead) is positioned over the macular hole, followed by additional Viscoat injection to achieve stabilization.

**Figure 3:**
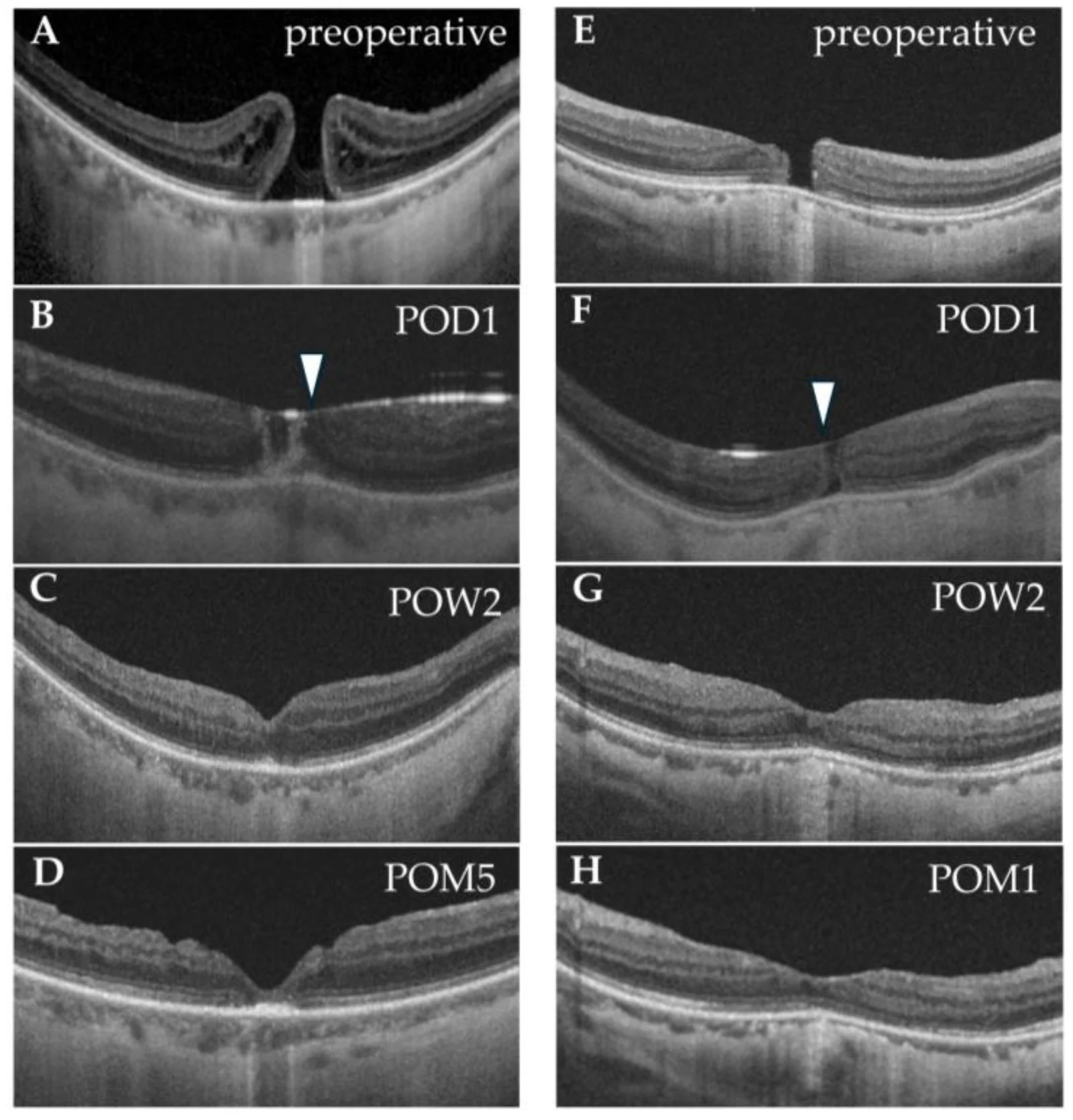
Optical coherence tomography (OCT) findings in the two cases. (**A**) Preoperative OCT of Case 1 demonstrates a full-thickness macular hole (MH). (**B**) One-day postoperative OCT in Case 1 shows the ILM flap covering the MH (arrowhead). (**C**) Two-week postoperative OCT in Case 1 confirms MH closure. (**D**) Five-month postoperative OCT in Case 1 shows sustained MH closure with persistent disruption of the external limiting membrane (ELM) and ellipsoid zone (EZ) beneath the fovea. (**E**) Preoperative OCT of Case 2 demonstrates a full-thickness MH. (**F**) One-day postoperative OCT in Case 2 shows the ILM flap covering the MH (arrowhead). (**G**) Two-week postoperative OCT in Case 2 confirms MH closure. (**H**) One-month postoperative OCT in Case 2 shows sustained MH closure with preservation of the ELM and EZ beneath the fovea.

## Data Availability

Data supporting the findings of this report are available from the corresponding author upon reasonable request.

## Supplementary Materials

The following supporting information can be downloaded at https://www.mdpi.com/article/10.3390/jcm15197392/s1, Supplemental Digital Content S1.mp4: Surgical procedure of the viscoelastic-assisted rescue technique for an unintended free internal limiting membrane flap. A dispersive ophthalmic viscosurgical device was used to capture, reposition, and stabilize the free ILM flap over the macular hole.
